# Multifunctional nanomaterials composed entirely of active pharmaceutical ingredients for synergistically enhanced antitumor and antibacterial effects

**DOI:** 10.3389/fphar.2024.1498728

**Published:** 2024-10-21

**Authors:** Qi An, Dongmei Wang, Liang Huang, Xiangyu Chen, Chuan Wang

**Affiliations:** ^1^ Scientific Reasearch and Teaching Department, Public Health Clinical Center of Chengdu, Chengdu, Sichuan, China; ^2^ National Engineering Research Center for Biomaterials, Sichuan University, Chengdu, China

**Keywords:** combination, curcumin, antibacterial, anticancer, all API

## Abstract

**Introduction:**

Multifunctional nanomaterials are emerging as promising tools for treating both cancer and bacterial infections. However, integrating dual therapeutic capabilities into a single system remains challenging. This study presents multifunctional nanoparticles (ECI-NPs) based on Epigallocatechin gallate (EGCG) oligomers, Curcumin (CUR), and Indocyanine Green (ICG) for combined cancer and bacterial treatment.

**Methods:**

ECI-NPs were synthesized via oxidative coupling of EGCG, CUR, and ICG. The nanoparticles were characterized for stability, size, drug loading, and release profiles. Cellular uptake, phototoxicity in melanoma cells, and antibacterial activity against *Escherichia coli* and *Staphylococcus aureus* were also evaluated.

**Results:**

ECI-NPs demonstrated optimal stability, high drug loading, and controlled release. Cellular studies showed increased uptake and greater phototoxicity in melanoma cells compared to free drugs. ECI-NPs also exhibited enhanced anticancer effects and strong antibacterial activity, outperforming the individual components.

**Discussion:**

The polyphenol-based ECI-NPs offer synergistic therapeutic effects, overcoming the limitations of free drugs in terms of solubility and efficacy. This dual-function platform shows potential for broader biomedical applications, addressing challenges in cancer and bacterial infections. Further research will focus on in vivo studies and clinical translation.

## 1 Introduction

The skin is the largest organ of the human body and serves as the first line of defense against external environmental threats (Yi, 2018). However, this exposure, particularly to ultraviolet (UV) radiation, can lead to carcinogenesis, with melanoma being one of the most aggressive and lethal forms of skin cancer (Bernard et al., 2019). “A study looking at UV exposure and genetic risks in relation to melanoma,” (Gandini, 2020). Melanoma arises from mutations in melanocytes and is associated with high rates of morbidity and mortality (Ossio et al., 2017; Schadendorf et al., 2018). Current clinical treatment strategies for early-stage melanoma primarily rely on surgical excision. Despite its effectiveness, surgery poses inherent limitations, including the inability to completely eradicate all tumor cells, which may lead to recurrence (Karaman and Alitalo, 2017). Moreover, postoperative bacterial infections at the surgical site can complicate wound healing, further exacerbating treatment challenges (Liu W.-S. et al., 2024; Wang et al., 2020). Consequently, the development of multifunctional materials that possess both anticancer and antibacterial properties has become a critical area of research in melanoma treatment. In recent years, phototherapy, particularly photodynamic therapy (PDT), has garnered attention due to its non-invasive and targeted nature, finding applications in the treatment of actinic keratosis, select skin cancers, and localized infections (Lu et al., 2024; Luo et al., 2024; Nguyen et al., 2022). PDT’s efficacy stems from the generation of reactive oxygen species (ROS) upon light activation of a photosensitizer, which leads to the destruction of cancer cells or bacteria. Therefore, the development of multifunctional biomaterials based on phototherapy strategies holds significant promise for both melanoma treatment and clinical translation (Hu et al., 2022; Huang et al., 2023; Xie et al., 2022).

Curcumin (CUR), a hydrophobic polyphenol derived from the rhizome of Curcuma longa, is well known for its broad-spectrum biological activities, including anti-inflammatory, antioxidant, antibacterial, and anticancer effects (Hasanzadeh et al., 2020; Liu X. et al., 2024). Despite its therapeutic potential, CUR’s clinical application has been hindered by its poor aqueous solubility and low bioavailability (Banerjee and Chakravarty, 2015; Xu et al., 2024). To overcome these limitations, nanomedicine delivery systems (NDS) have emerged as a promising strategy. By encapsulating or loading CUR into nanocarriers, NDS can enhance its solubility, stability, and bioavailability, thus improving its therapeutic efficacy (Feltrin et al., 2022; Wang et al., 2015; Zhang et al., 2022). These nanomedicine systems have shown significant potential in both anticancer and antibacterial treatments. For example, functionalized zinc oxide nanoparticles with chitosan have been employed as CUR delivery systems, resulting in multifunctional nanomaterials that exhibit enhanced anticancer activity and lower minimum inhibitory concentrations (MIC) compared to their individual components. Furthermore, cyclodextrin-based hollow spheres encapsulating CUR have demonstrated increased cytotoxicity and antibacterial activity.

Indocyanine Green (ICG) is a near-infrared fluorescent dye with advantages like good biocompatibility and low toxicity, but it has several limitations (Phillips and Finley, 2020; Porcu et al., 2016; Portnoy et al., 2016). These include poor photostability, low water solubility, non-specific distribution in the body, limited fluorescence quantum yield, and rapid metabolism. To overcome these challenges, researchers are developing ICG-based nanocarriers and modified derivatives to improve its stability, targeting capability, and imaging performance (Gu et al., 2024; Kwon et al., 2023; Lajunen et al., 2018). CUR and resveratrol exhibit a synergistic effect when combined. Hang Hu et al. developed a multifunctional chemotherapy-PDT nanoplatform co-loaded with curcumin (CUR) and indocyanine green (ICG) (Hu et al., 2023). Their study demonstrated that CUR inhibited tumor angiogenesis, thereby enhancing the PDT efficacy of ICG *in vitro*. Similarly, Siqi Zhang and colleagues designed a smart multimodal composite material for combating bacterial infections, where the incorporation of CUR effectively compensated for the limitations of ICG in inhibiting resistant bacteria, highlighting synergistic therapeutic effects (Zhang et al., 2022). However, an excessive amount of carrier entering the body may pose potential risks. The “all API” approach offers several advantages, including higher drug loading efficiency, reduced use of excipients, improved stability, and enhanced pharmacokinetics. It simplifies manufacturing, decreases the risk of side effects, and allows for more precise drug delivery, ultimately improving therapeutic efficacy while minimizing risks and costs (Fang and Chen, 2024; Liu and Zhang, 2022). In our previous work, we developed a polyphenol-based nano drug delivery system capable of simultaneously delivering multiple poorly soluble molecules, addressing the solubility issues of various drugs and enhancing therapeutic efficacy (Chen et al., 2023; Chen et al., 2020; Tong et al., 2023; Tong et al., 2024; Tong et al., 2021; Yi et al., 2020a; Yi et al., 2023).

**SCHEME 1:**
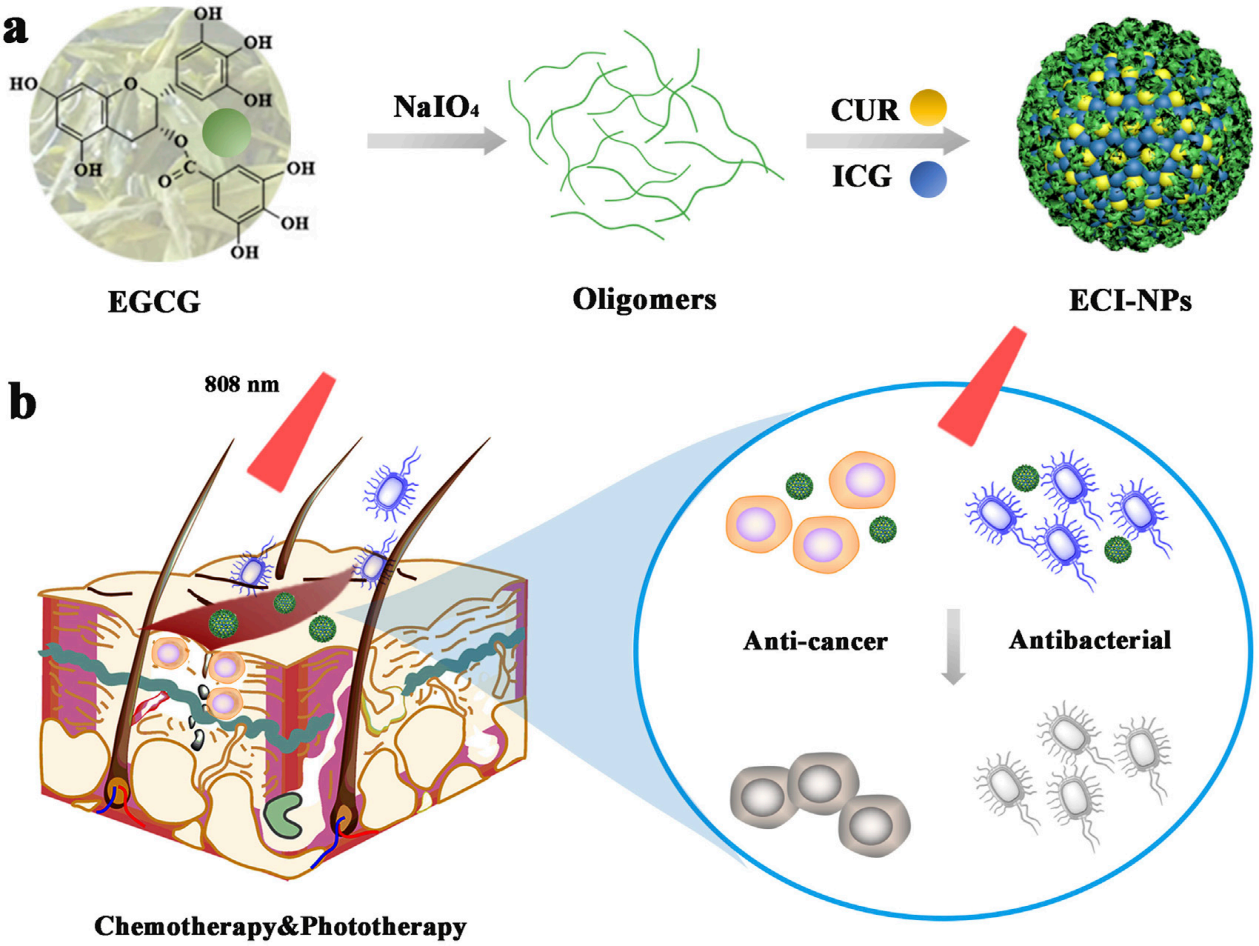
**(A)** Schematic representation of nanoparticles (ECI-NPs) formed by EGCG oligomers, chemotherapeutic drug curcumin (CUR), and photosensitizer indocyanine green (ICG). **(B)** ECI-NPs exhibit the potential to kill tumor cells and bacteria through near-infrared light-induced chemo-phototherapy, indicating their therapeutic potential for postoperative infected wounds in tumor treatment.

In the present study, we developed a tea polyphenol-based nanocarrier using oxidative coupling reactions to simultaneously load CUR and ICG, constructing a multifunctional nanomaterial (Scheme 1). This material was designed to achieve dual anticancer and antibacterial effects through phototherapy for melanoma treatment. Specifically, we employed the non-covalent interactions between EGCG oligomers, ICG, and CUR to construct multifunctional nanoparticles (ECI-NPs). The resulting nanoparticles not only improved the solubility and stability of CUR but also enhanced its therapeutic efficacy through synergistic photodynamic mechanisms, offering potential for simultaneous anticancer and antibacterial therapy. Initially, we characterized the physicochemical properties of ECI-NPs, including particle size, zeta potential, drug loading efficiency, and drug release profiles. The results indicated that the nanoparticles exhibited favorable stability and high drug loading capacity. Subsequent biological evaluations were conducted at the cellular level, including assessments of cellular uptake, phototoxicity, and anticancer efficacy. Compared to free drugs, ECI-NPs demonstrated superior cellular uptake and enhanced phototherapy-induced cytotoxicity. Additionally, ECI-NPs exhibited significantly greater anticancer activity against melanoma cells. Antibacterial assays against *Escherichia coli* and *Staphylococcus aureus* further demonstrated that ECI-NPs had stronger antibacterial effects than free CUR and ICG. In conclusion, our study provides preliminary evidence for the feasibility of ECI-NPs as a multifunctional nanomaterial with both anticancer and antibacterial properties. The combined effects of EGCG oligomers, CUR, and ICG within the nanoparticle structure allowed for effective eradication of tumor cells and bacteria under phototherapeutic conditions. Future studies will focus on further evaluating the safety and efficacy of this nanomaterial *in vivo* models to support its clinical potential for melanoma treatment and bacterial infection control.

## 2 Materials and methods

### 2.1 Experimental reagents

Epigallocatechin gallate (EGCG, ≥92%, Sun Green Treasure Co., Ltd., Wuxi, China); sodium periodate (NaIO₄, Chengdu Cologne Chemical Co., Ltd., China); curcumin (CUR, TCI Development Co., Ltd., Shanghai, China); indocyanine green (ICG, Meilun Biological Technology Co., Ltd., Dalian, China); 1,3-diphenylisobenzofuran (DPBF, Adamas Reagent Co., Ltd., Shanghai, China); Cell Counting Kit-8 (CCK-8, Dojindo Laboratories, Japan); peptone and yeast extract (Oxoid, UK); agarose (Solarbio Life Sciences, Beijing, China).

### 2.2 Preparation of ECI-NPs

EGCG (600 mg) was dissolved in 100 mL of water, and the pH of the solution was adjusted to approximately two using hydrochloric acid. Sodium periodate (NaIO₄, 120 mg) was added to the solution and allowed to react at room temperature for 1 h, yielding EGCG nanoparticles (E-NPs). Subsequently, E-NPs were mixed with 50 μL of CUR ethanol solution (10 mg mL⁻^1^) and vortexed for 5 s. Finally, the mixture was added to 1 mL of ICG aqueous solution (500 μg mL⁻^1^), followed by three washes with saline to obtain multifunctional EGCG nanoparticles (ECI-NPs). EC-NPs, loaded with CUR, were prepared by replacing the ICG aqueous solution with pure water. Different formulations of EC-NPs and ECI-NPs were prepared by adjusting the amounts of E-NPs, CUR, and ICG in the system.

### 2.3 Characterization of ECI-NPs

The morphology of E-NPs, EC-NPs, and ECI-NPs was observed by transmission electron microscopy (TEM). The hydrodynamic size and zeta potential of the particles were measured using dynamic light scattering (DLS). A sufficient amount of freeze-dried nanoparticles was mixed with potassium bromide powder, ground, pressed into pellets, and analyzed by Fourier-transform infrared (FTIR) spectroscopy in the range of 400–4,000 cm⁻^1^. The ultraviolet (UV) spectra and fluorescence emission spectra of EC-NPs and ECI-NPs were recorded using a UV-Vis spectrophotometer and a near-infrared fluorescence spectrometer, respectively.

### 2.4 *In Vitro* photodynamic and photothermal properties of ECI-NPs

ICG and ECI-NPs (equivalent ICG concentration: 10 μg mL⁻^1^) were mixed with 50 μL of DPBF solution (1.5 mg mL⁻^1^, acetonitrile) in 2 mL of water. The mixture was irradiated with an 808 nm laser at a power density of 2 W cm⁻^2^, and the absorbance at 420 nm was recorded every 5 min. Temperature changes in ICG and ECI-NPs were recorded under laser irradiation at power densities of 1, 1.5, or 2 W cm⁻^2^. The heating curves of ECI-NPs at different concentrations, as well as their heating/cooling cycle curves, were also evaluated.

### 2.5 Cell experiments

For cellular uptake experiments, B16 melanoma cells were seeded in six-well plates and grown to approximately 80% confluency. CUR, ICG, CUR/ICG, EC-NPs, and ECI-NPs (equivalent CUR and ICG concentrations of five and 2.5 μg mL⁻^1^, respectively) were added to the wells, and cells were incubated for 4 h. The cells were then collected and analyzed using flow cytometry. The fluorescence of CUR and ICG was detected in the BV421 and APC-Cy7 channels, respectively. HCT116 and B16 cells were seeded in 96-well plates at a density of 5 × 10³ cells per well. After 24 h, the cells were treated with various concentrations of EC-NPs and ECI-NPs, followed by laser irradiation (3 W cm⁻^2^, 3 min) after 6 h of incubation. Cell viability was assessed 24 h post-irradiation using a CCK-8 assay, and absorbance at 450 nm was measured to calculate cell viability.

### 2.6 Bacterial experiments

Single colonies of *S*. *aureus* and *E*. *coli* were selected using a sterile inoculation loop and cultured in 5 mL of liquid medium at 37°C in a shaker. Bacterial suspension absorbance at 600 nm was measured using a microplate reader. An absorbance of one corresponded to bacterial concentrations of 6 × 10⁸ CFU mL⁻^1^ for *E. coli* and 1.5 × 10⁹ CFU mL⁻^1^ for *S. aureus*. Bacterial suspensions were diluted to approximately 10⁴ CFU mL⁻^1^ based on the absorbance-concentration ratio. CUR, ICG, EC-NPs, and ECI-NPs (equivalent CUR and ICG concentrations of 500 and 250 μg mL⁻^1^, respectively) were dispersed in sterile PBS and incubated with equal volumes of bacterial suspension for 24 h. ICG and ECI-NPs were irradiated with a laser at 3 W cm⁻^2^ for 10 min. PBS without nanoparticles was used as the control. After incubation, 10 μL of the bacterial solution was plated on agar plates, incubated for 12 h, and bacterial colony counts were determined to assess bacterial viability.

## 3 Results and discussion

**FIGURE 1:**
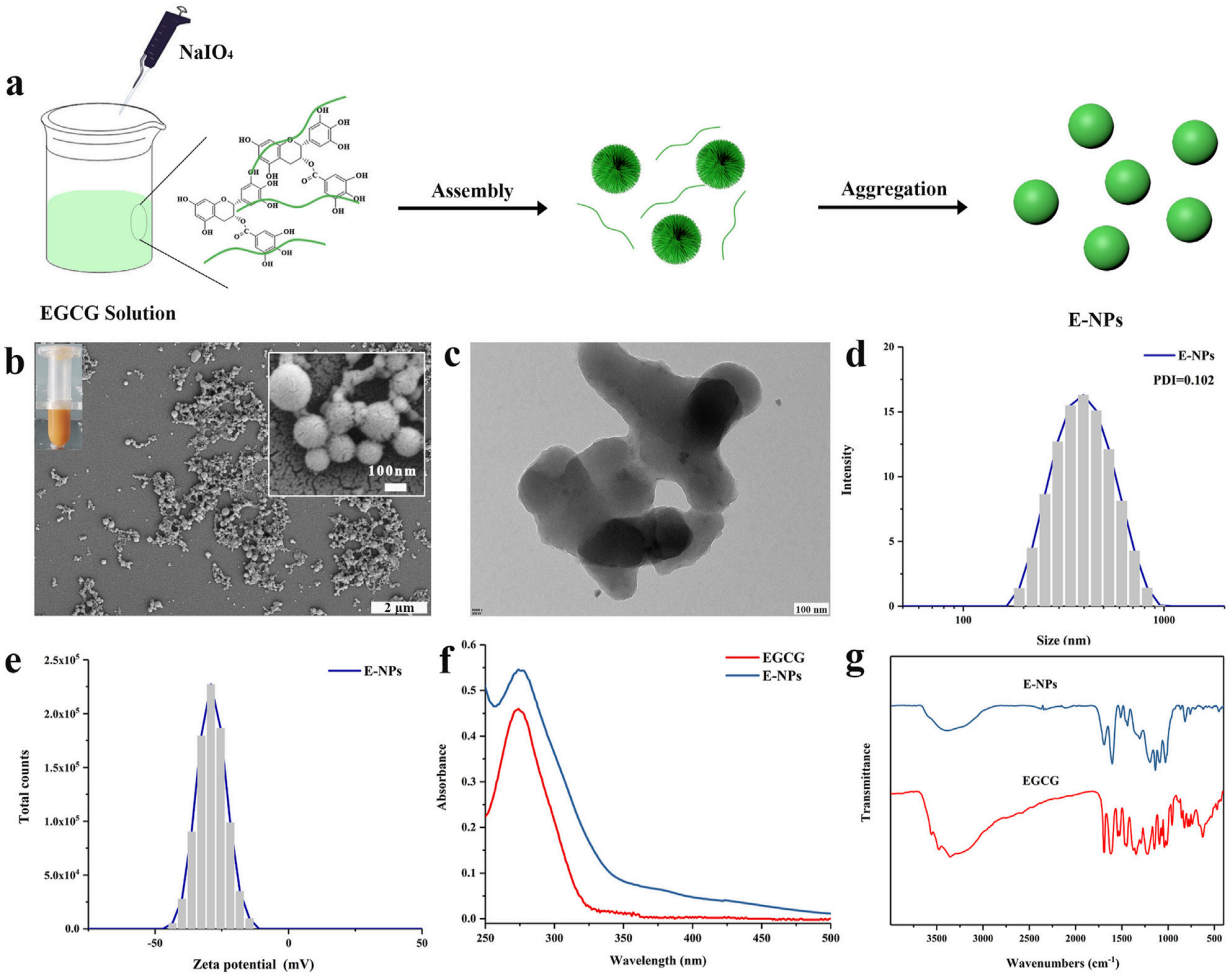
Physicochemical characterization of tea polyphenol nano-oligomers (E-NPs): **(A)** Schematic illustration of synthesis; **(B)** Scanning electron microscope (SEM) image, with a digital image of E-NPs in the inset; **(C)** Transmission electron microscope (TEM) image; **(D)** Hydrodynamic size distribution; **(E)** Zeta potential distribution; **(F)** UV-Vis spectrum; **(G)** Infrared spectrum.

Tea polyphenol nano-oligomers (E-NPs) were successfully prepared using oxidative coupling technology under acidic conditions. As shown in Figure 1A, sodium periodate was gradually added dropwise into a tea polyphenol solution with a pH of two at room temperature. The resulting polyphenol oligomers self-assembled into nanoparticles through non-covalent interactions. Figure 1B presents the SEM image of the prepared E-NPs, where noticeable particle aggregation can be observed. SEM images at higher magnification show that the nanoparticles are mainly spherical, but the size uniformity is relatively poor. When dispersed in aqueous solution, E-NPs appear dark yellow, likely due to the oxidation of the tea polyphenols. As depicted in Figure 1C, the E-NPs exhibit irregular nanoparticle morphology. The hydrodynamic size distribution (Figure 1D) reveals an average particle size of approximately 396 nm with a polydispersity index (PDI) of 0.102, indicating relatively uniform particle size distribution. However, zeta potential measurements show a surface charge of −29.09 mV, suggesting poor stability of the particles in water. The UV-Vis spectrum (Figure 1F) shows that the absorption peak of the E-NPs coincides with that of EGCG, confirming that EGCG is the primary component of the nanoparticles. The infrared spectrum provides key insights into the molecular changes during the oxidation and polymerization of EGCG. Firstly, the broadening of the peak at 3,400 cm⁻^1^ indicates O-H stretching vibrations, reflecting the formation of hydrogen bonds and increased hydroxyl group interactions due to polyphenol oxidation. This broadening suggests enhanced intramolecular or intermolecular hydrogen bonding, contributing to the stability and self-assembly of nanoparticles. Secondly, the appearance of the absorption peak at 1,143 cm⁻^1^ is attributed to the formation of ether bonds (C-O-C) through oxidative coupling reactions. Additionally, in the EGCG sample, the strong absorption peak in the 500–847 cm⁻^1^ range, associated with out-of-plane C-H bond deformation in the benzene ring, disappears in EC particles, indicating reduced deformation due to cross-linking. The absorption peak at 1,693 cm⁻^1^, characteristic of carbonyl stretching in EGCG, is significantly reduced in colloidal spheres, further confirming extensive polymerization of EGCG (Figure 1G). In conclusion, these experimental results demonstrate that under acidic conditions, the use of a strong oxidizing agent can rapidly induce the oxidation and self-polymerization of tea polyphenols, leading to the formation of negatively charged nanoparticle aggregates. This method provides a simple and efficient approach for the design and development of polyphenol-based nanomaterials, which may have broad potential applications in the biomedical field.

**FIGURE 2:**
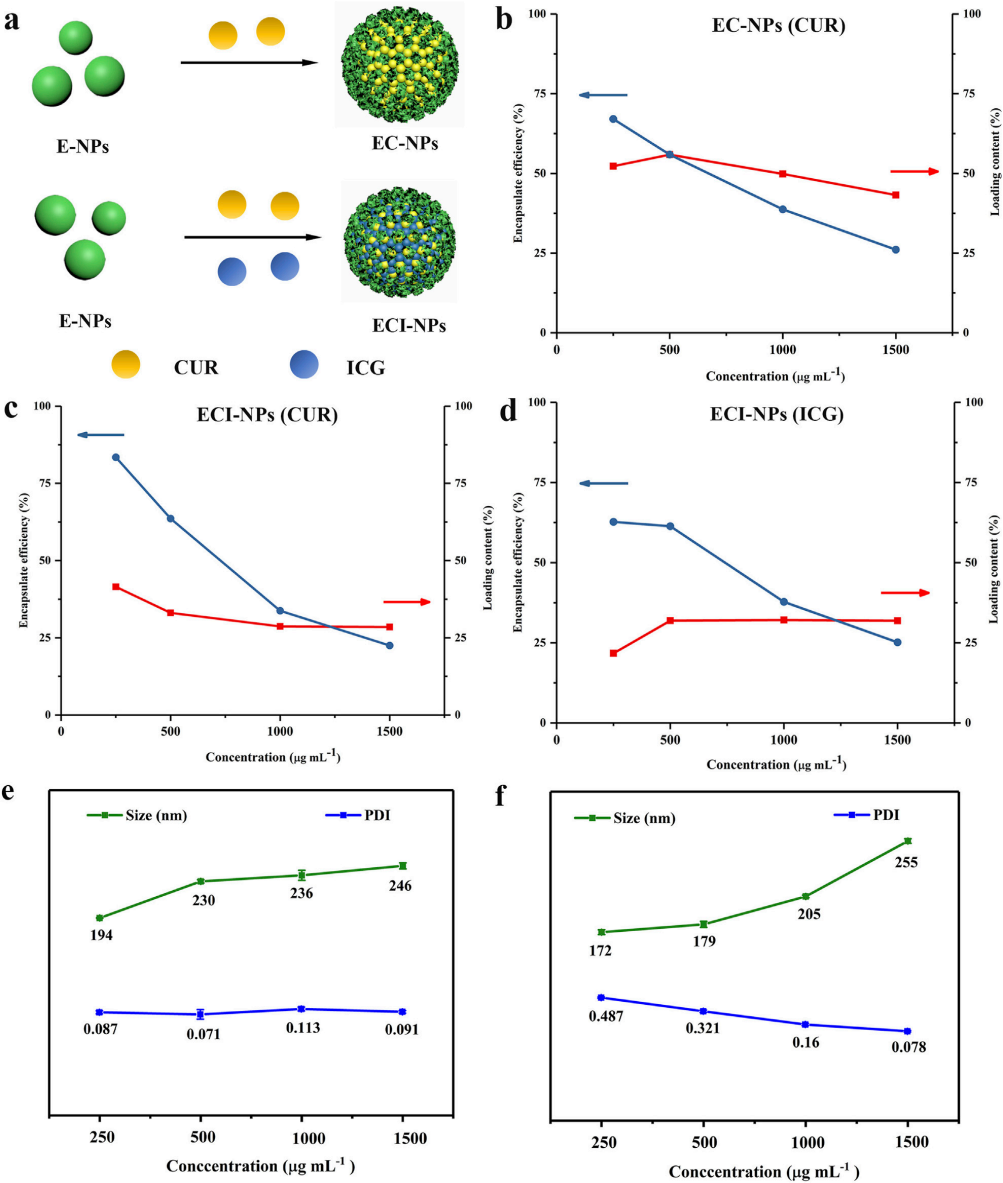
**(A)** Schematic illustration of the synthesis of multifunctional tea polyphenol nanomedicines. **(B)** Drug loading and encapsulation efficiency of CUR alone. Variations in encapsulation efficiency (EE) and drug loading (DL) of **(C)** CUR and **(D)** ICG in ECI-NPs, with CUR or ICG concentrations kept constant while changing the EC concentration. **(E)** Hydrodynamic size and **(F)** polydispersity index (PDI) of EC-NPs and ECI-NPs.

In previously reported studies, polyphenol-based oxidative coupling technology has been used to prepare porous microspheres, artificial melanin nanoparticles, and pH-responsive polyphenol nanoparticles (Chen et al., 2013; Zhou et al., 2019). However, there are relatively few reports on using oxidative coupling technology to construct multifunctional nanodrug carriers. In this study, the drug-loading capacity of E-NPs for CUR and ICG was investigated. Initially, the prepared E-NPs were dissolved in DMSO along with CUR to form a mixed solution, which was subsequently added to 1 mL of aqueous ICG solution. After centrifugation, tea polyphenol nanoparticles co-loaded with CUR and ICG (ECI-NPs) were obtained. As a control, EC-NPs, tea polyphenol nanoparticles loaded solely with CUR, were prepared using deionized water, with the synthesis schematic shown in Figure 2A. In a 1 mL system, the concentration of the polyphenol carrier was varied to achieve different formulations of EC-NPs and ECI-NPs. As shown in Figure 2B, as the carrier concentration increased from 250 μg mL⁻^1^–1,500 μg mL⁻^1^, the encapsulation efficiency of EC-NPs for CUR decreased, while the drug-loading capacity remained relatively constant. The maximum drug-loading capacity and encapsulation efficiency were 55.9% and 67.1%, respectively. Figures 2C, D depict the changes in drug-loading capacity and encapsulation efficiency of ECI-NPs for CUR and ICG at different carrier concentrations. Similarly, with increasing carrier concentration, the encapsulation efficiency for both CUR and ICG in ECI-NPs gradually decreased, whereas the drug-loading capacity remained stable. Additionally, ECI-NPs exhibited comparable drug-loading and encapsulation efficiency for both CUR and ICG. Particle size analysis revealed that as the carrier concentration increased, the particle size of EC-NPs grew from 194 nm to approximately 246 nm, while the polydispersity index (PDI) remained around 0.1 (Figure 2E). Compared to E-NPs, EC-NPs showed a significant reduction in particle size, with no change in PDI, indicating that the CUR-loaded EC-NPs improved the water dispersibility and stability of CUR. Upon the introduction of ICG, ECI-NPs exhibited smaller particle sizes at the same carrier concentration (Figure 2F), although their water dispersibility slightly decreased, as reflected by a higher PDI compared to E-NPs and EC-NPs. In summary, the synthesized tea polyphenol nano-oligomers (E-NPs) successfully enabled dual loading of CUR and ICG, and the resulting EC-NPs and ECI-NPs exhibited smaller particle sizes. Based on these findings, EC-NPs and ECI-NPs prepared at carrier concentrations of 500 and 1,500 μg mL⁻^1^ were selected for subsequent experiments.

**FIGURE 3:**
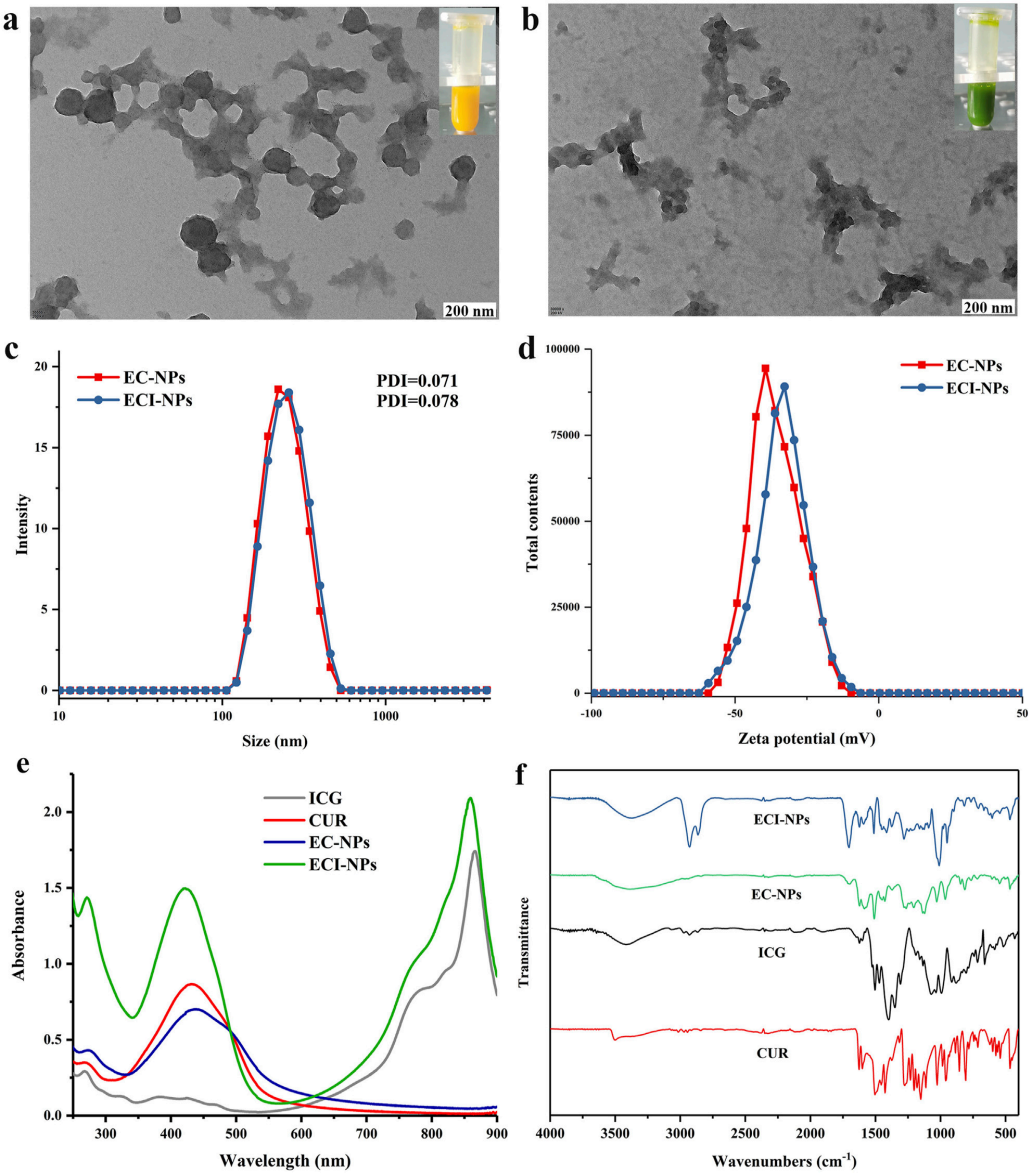
Transmission electron microscopy (TEM) images of **(A)** EC-NPs and **(B)** ECI-NPs; insets show corresponding digital photographs. **(C)** Hydrodynamic size distribution and **(D)** zeta potential distribution of EC-NPs and ECI-NPs. **(E)** Ultraviolet-visible (UV-Vis) absorption spectra and **(F)** Fourier-transform infrared (FTIR) spectra of ECI-NPs.

As shown in Figure 3A, EC-NPs are spherical nanoparticles, and their aqueous dispersion appears yellow. In contrast, ECI-NPs form amorphous nanoparticle aggregates with a green-colored aqueous dispersion (Figure 3B). The introduction of ICG into the system disrupts the spherical morphology of EC-NPs, likely due to the enhanced non-covalent interactions induced by the small organic molecule ICG. Moreover, the colors of the aqueous dispersions are derived from the yellow CUR and the green ICG. The particle size distribution curves of EC-NPs and ECI-NPs in water are almost identical, with an average particle size of approximately 250 nm and PDI values of 0.071 and 0.078, respectively (Figure 3C), indicating good dispersibility in water. As shown in Figure 3D, both EC-NPs and ECI-NPs exhibit negative surface charges, consistent with previous results for tea polyphenol-based nanoparticles. The negative surface charge is hypothesized to originate from the multiple phenolic hydroxyl groups in the tea polyphenol structure. The UV spectra of free drugs and nanoparticles are shown in Figure 3E. The characteristic absorption peaks of ICG and CUR are observed at 880 nm and 440 nm, respectively. The strongest absorption peak of EC-NPs appears around 440 nm, confirming the successful loading of CUR in EC-NPs. For ECI-NPs, two strong absorption peaks are observed at 440 nm and 880 nm, indicating the co-loading of both CUR and ICG. These UV spectrum data confirm that the tea polyphenol-based oligomers successfully encapsulated both CUR and ICG at the nanoscale. In Figure 3F, the infrared spectra of both EC-NPs and ECI-NPs exhibit the stretching vibration peak of the ether bond C-O at 1,280 cm⁻^1^, characteristic of CUR, further verifying the effective encapsulation of the poorly soluble polyphenol CUR by the tea polyphenol-based oligomers. Additionally, the peak at 1,092 cm⁻^1^ in the infrared spectrum of ECI-NPs is attributed to the sulfonyl group of ICG. The UV and infrared spectra together confirm the successful loading of CUR and ICG by E-NPs.

**FIGURE 4:**
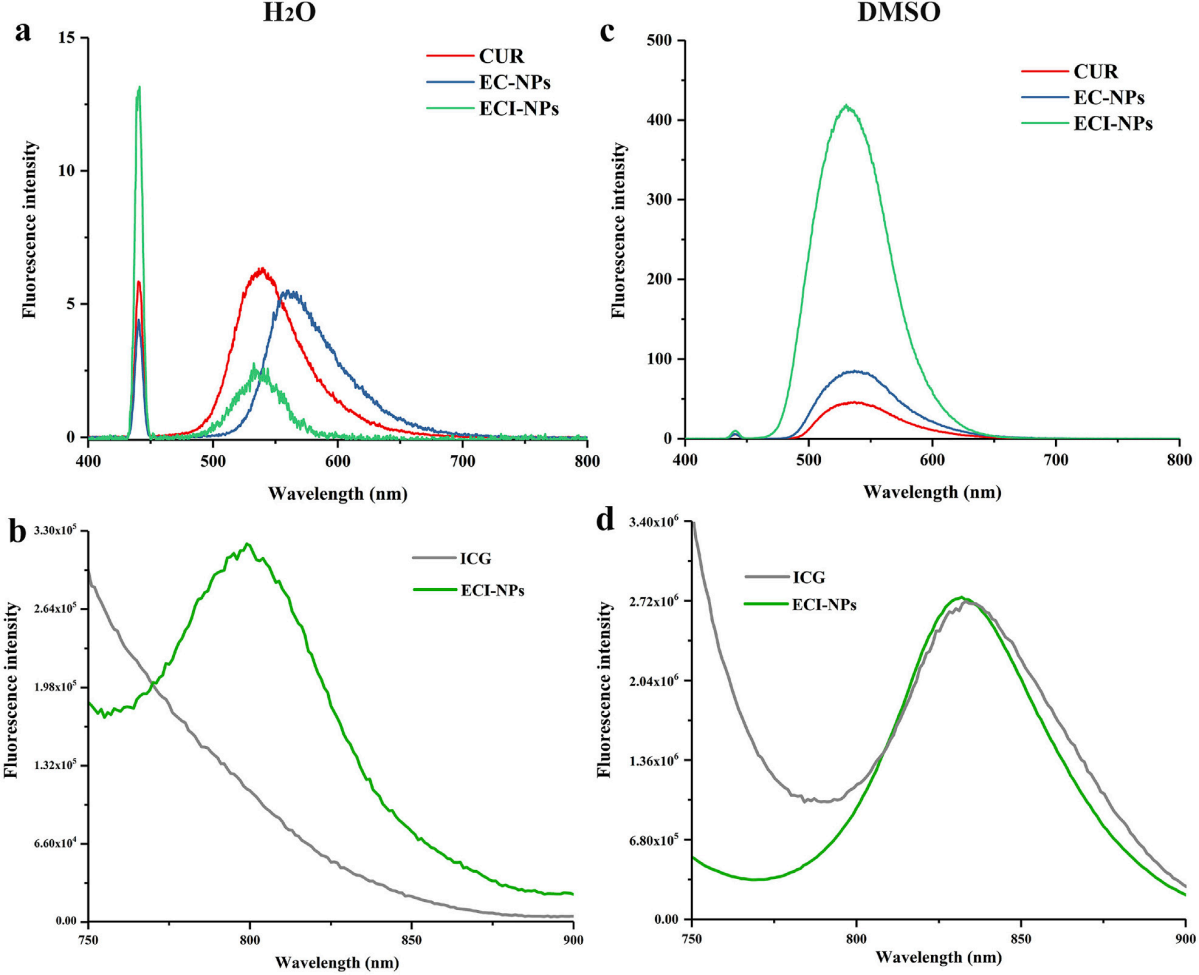
Fluorescence emission spectra of **(A, B)** EC-NPs and **(C, D)** ECI-NPs in water and dimethyl sulfoxide (DMSO), illustrating their optical properties and solvent effects.

Fluorescence emission spectra were used to investigate the drug-loading behavior of EC-NPs and ECI-NPs. As shown in Figure 4A, under 440 nm excitation, the emission peak of CUR in water is located around 530 nm, whereas a red shift occurs for EC-NPs, indicating strong non-covalent interactions between EGCG oligomers and CUR. After the introduction of ICG, these interactions are weakened, resulting in no significant shift in the emission peak for ECI-NPs. Additionally, the emission intensity of EC-NPs and ECI-NPs is lower than that of CUR alone, which contrasts with the spectra obtained in DMSO (Figure 4B). This phenomenon has been observed in previous studies and is consistent with the fluorescence shielding effect observed in nanocarriers due to drug encapsulation (Yi et al., 2020b). Figures 4C, D shows the fluorescence emission spectra of ICG and ECI-NPs under different solvents, with excitation wavelengths set at 680 nm. Although ICG, as a photosensitizer, is an amphiphilic small molecule that can disperse well in water, its optical properties are influenced by the solvent environment. In water, ECI-NPs exhibit an emission peak at 800 nm, while ICG does not. In DMSO, ECI-NPs display an emission peak around 840 nm, consistent with ICG. These results suggest that ECI-NPs improve the dispersibility of ICG in water, alleviating self-quenching at high concentrations, enhancing quantum yield, and demonstrating potential as a nanocarrier for ICG delivery.

Upon near-infrared (NIR) irradiation, ICG not only produces reactive oxygen species (ROS) but also converts a significant portion of absorbed light into heat. To validate the multifunctional potential of ECI-NPs as a platform, their photodynamic and photothermal properties were investigated, with pure water serving as the control group. DPBF was used as a singlet oxygen probe, and the fluorescence intensity at 450 nm was recorded, with results shown in Figure 6. 8.Within 15 min of irradiation at 808 nm, the fluorescence intensity of all groups decreased by approximately 10%. After 30 min of irradiation, the fluorescence intensity of the control group decreased to around 83%, while the ICG and ECI-NPs groups exhibited lower intensity ratios of 58% and 43%, respectively, confirming the photodynamic activity of both. Notably, the ECI-NPs group showed a lower intensity ratio than the ICG group, indicating that ECI-NPs generated more singlet oxygen after irradiation, leading to the formation of more internal peroxides with DPBF and a more pronounced decrease in fluorescence intensity. These results suggest that ECI-NPs exhibit superior photodynamic activity compared to ICG.

**FIGURE 5:**
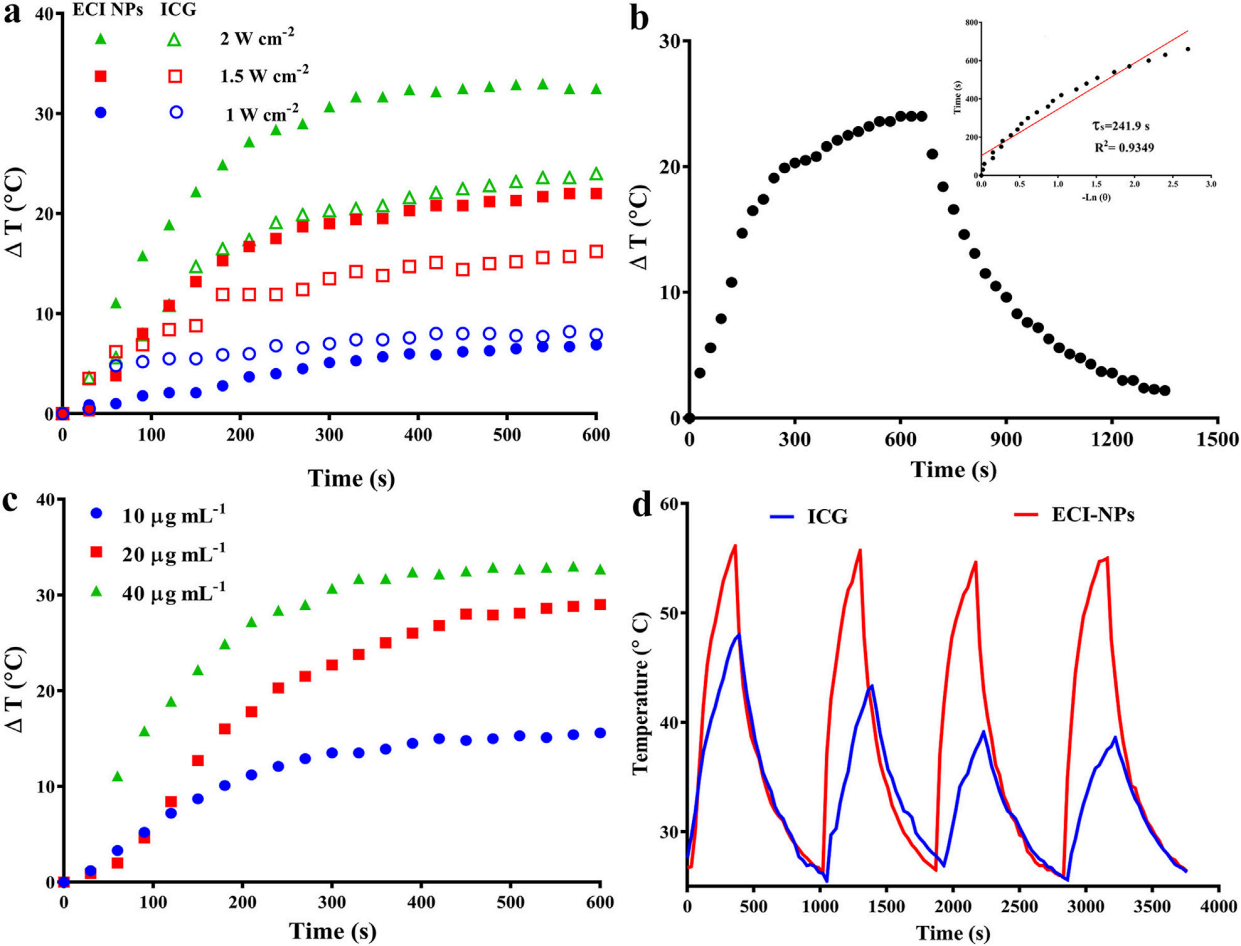
**(A)** Photothermal heating curves of ICG and ECI-NPs under different power densities, demonstrating photothermal conversion efficiency. **(B)** Heating/cooling curves of ECI-NPs, with inset showing the fitted linear relationship between time and temperature during the cooling process. **(C)** Heating curves of ECI-NPs at different concentrations, indicating the concentration-dependent heating rate. **(D)** Heating/cooling cycle curves of ICG and ECI-NPs, comparing their photothermal stability.

The photothermal conversion efficiency of ECI-NPs was also studied, and the results are shown in Figure 5. First, the temperature-time curves of ICG and ECI-NPs were recorded under different irradiation doses. As irradiation time and power increased, the temperatures of both ICG and ECI-NPs rose significantly. At a power of 1 W cm⁻^2^, the temperature increase of ICG and ECI-NPs was similar, with temperature rises of 6.9°C and 7.9°C, respectively, after 10 min of irradiation. When the power was increased to 2 W cm⁻^2^, the temperature rises of ICG and ECI-NPs were 16.2°C and 22°C, respectively, indicating that ECI-NPs exhibited higher photothermal conversion efficiency. At a power of 2 W cm⁻^2^, the temperature increases for ICG and ECI-NPs were 24°C and 32.5°C, respectively, showing that the temperature rise of ECI-NPs was more pronounced with increasing power. Subsequently, the photothermal conversion efficiency of ECI-NPs was calculated based on the temperature-time curve after irradiation, with a slope (τ) of 241.9 s obtained (Figure 5B). The photothermal conversion efficiency of ECI-NPs was calculated to be 20.21%, indicating that ECI-NPs improve the photothermal conversion efficiency of ICG, thereby enhancing its potential for photothermal applications.

Additionally, the temperature increase of ECI-NPs under different concentrations with laser irradiation was investigated (Figure 5C). When the concentration increased from 10 μg mL⁻^1^–20 μg mL⁻^1^, the maximum temperature rise of ECI-NPs increased from 15.6°C to 29°C, a 13.4°C improvement. Further increases in concentration resulted in a less pronounced temperature rise, with only a 3.7°C increase. Finally, the photothermal stability of ICG and ECI-NPs was examined. The temperature increase and decrease curves over four heating/cooling cycles are shown in Figure 5D. The temperature rise for the ICG group decreased with each cycle, likely due to photobleaching. In contrast, ECI-NPs maintained consistent temperature increases across all cycles, demonstrating good photothermal conversion efficiency. These results suggest that ECI-NPs improve the photostability and photodynamic/photothermal performance of ICG, making them a superior multifunctional nanoplatform for potential applications.

**FIGURE 6:**
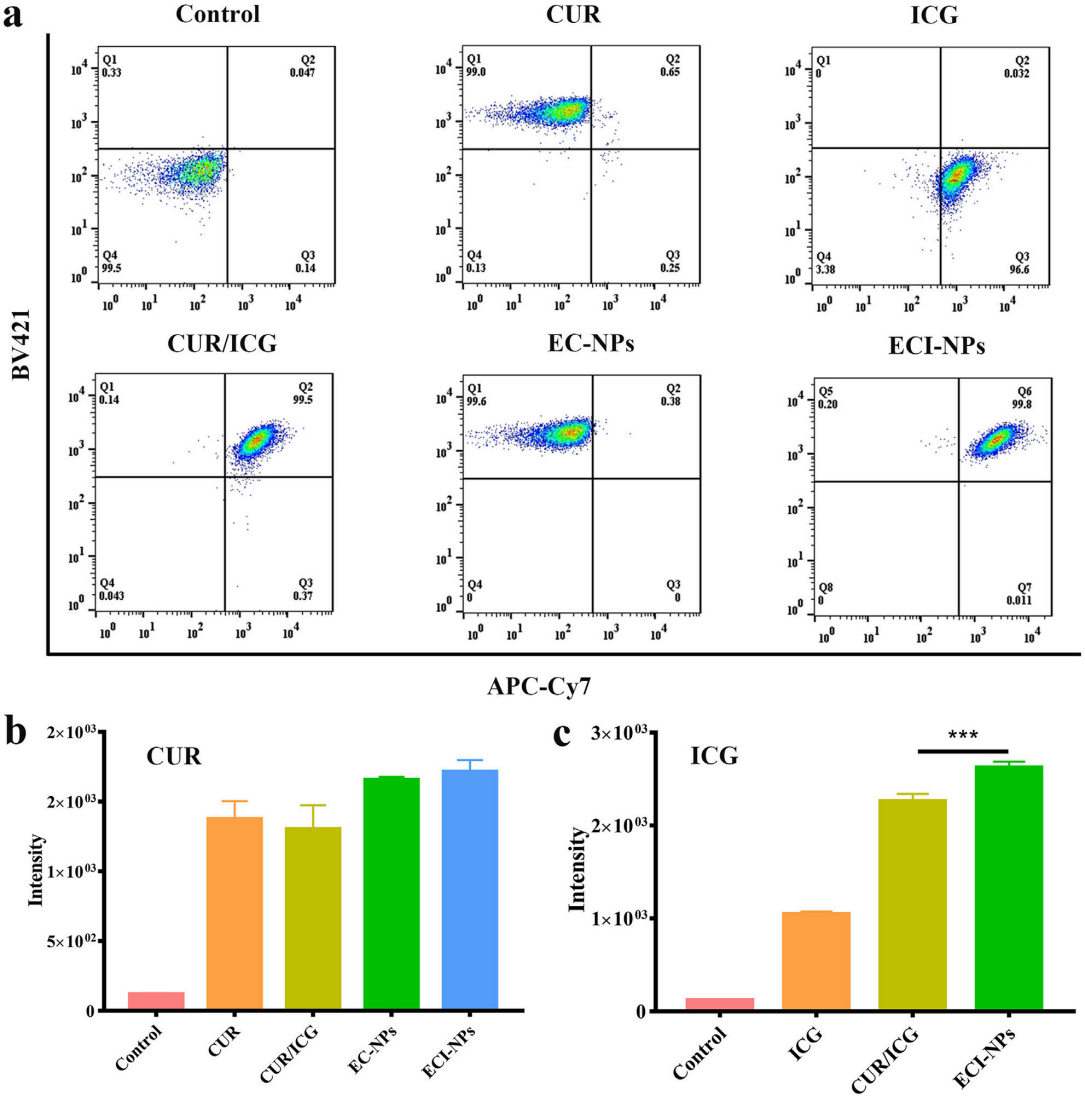
Flow cytometry analysis of B16 melanoma cells after treatment with nanoparticles for various durations. **(A)** Intracellular signal scatter plot, showing nanoparticle distribution and accumulation within cells. **(B)** Fluorescence intensity analysis; CUR equivalent at 5 μg mL⁻^1^. **(C)** Fluorescence intensity analysis; ICG equivalent at 2.5 μg mL⁻^1^.

The preceding results validated the photothermal and photodynamic capabilities of ECI-NPs. Next, we investigated their antitumor activity through cellular experiments. Initially, we assessed the fluorescence signals of BV421 and APC-Cy7 channels in tumor cells after treatment with the materials. As shown in Figure 6A, the scatter plots for EC-NPs and CUR groups in the BV421 channel were similar, indicating that B16 cells exhibited comparable internalization capabilities for both EC-NPs and CUR. The fluorescence intensity of the ECI-NPs group in this channel was also comparable to the other two groups, suggesting that the oligomeric tea polyphenol nanocarriers did not significantly affect the cellular uptake of encapsulated CUR. In the APC-Cy7 channel, the fluorescence uptake in the CUR/ICG group was substantially higher than in the ICG group, which contrasts with the BV421 channel results. CUR/ICG did not impact CUR uptake by tumor cells but enhanced cellular uptake of ICG, likely due to the increased cellular uptake of amphiphilic ICG facilitated by CUR’s lipophilicity. As depicted in Figures 6B, C, the intracellular fluorescence intensity of the ECI-NPs group was significantly higher than that of the ICG and CUR/ICG groups, indicating that nanomization of CUR and ICG using E-NPs confers excellent cellular uptake ability, with ECI-NPs showing superior ICG internalization.

**FIGURE 7:**
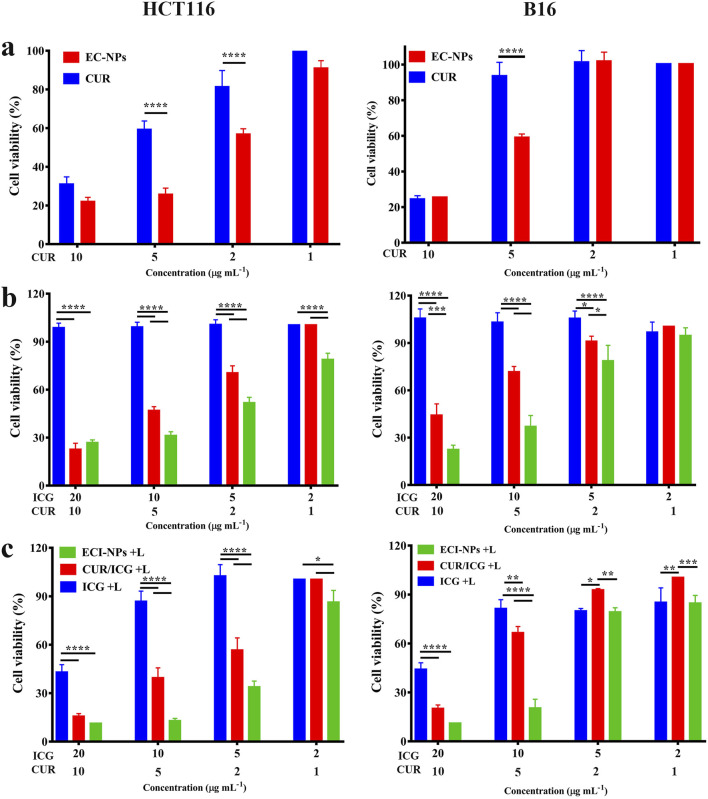
**(A)** Cell viability of HCT116 cells after 24 h of treatment with EC-NPs. **(B)** Cell viability of B16 cells after 24 h of treatment with EC-NPs under non-irradiated and **(C)** irradiated conditions, demonstrating the photosensitizing activity of the nanoparticles.

Cell toxicity evaluations were conducted using HCT116 and B16 cells Figure 6. 11 presents the cell viability after 24 h of incubation with each material. Compared to the CUR group, the HCT116 and B16 cell survival rates were lower in the EC-NPs group (Figure 7A), demonstrating strong antitumor activity of EC-NPs. Given the minimal difference in intracellular fluorescence signals between CUR and EC-NPs, it is inferred that the enhanced tumor cell suppression arises from the tea polyphenols in the particles. In the absence of light, the cell survival rate in the ICG group remained near 100%, whereas CUR/ICG and ECI-NPs groups exhibited significant cytotoxicity (Figure 7B). Notably, the cell survival rate in the ECI-NPs group was lower than that in the CUR/ICG group, indicating that besides CUR’s inhibitory effect, the tea polyphenols in ECI-NPs also augment the particles’ antitumor activity. Additionally, the EC-NPs group showed a lower cell survival rate compared to the CUR/ICG group, further suggesting that tea polyphenols as nanocarriers not only nanomize the payload (i.e., CUR and ICG) but also exhibit antitumor activity, enhancing the biological efficacy of the constructed nanodelivery system. The lower tumor cell survival rate in the ECI-NPs group compared to the EC-NPs group in the absence of light may be attributed to the lower CUR loading in ECI-NPs, resulting in higher intracellular tea polyphenol content and, consequently, stronger suppression.Figure 7C shows that after light exposure, all material groups containing ICG exhibited a decrease in cell survival rate, indicating their antitumor phototoxicity. Compared to the ICG group, the CUR/ICG group, due to the combined chemotherapeutic effect of CUR, showed even lower tumor cell viability, demonstrating that the combination of CUR and ICG can enhance the suppression of tumor cells. Among all experimental groups, the ECI-NPs group exhibited the lowest cell survival rate post-light exposure, confirming its potent biological activity. After high-concentration ECI-NPs treatment, the tumor cell survival rate was around 10%. This pronounced antitumor effect is attributed to the high intracellular ICG content in the ECI-NPs group, as well as the combined chemotherapeutic effects of CUR and tea polyphenols. The similar trend observed in cell toxicity experiments with B16 and HCT116 cells preliminarily validates the broad-spectrum antitumor activity of ECI-NPs. Multidrug resistance is a major cause of chemotherapy failure, while phototherapy is less likely to induce resistance. Therefore, combined chemotherapy and phototherapy is a promising multimodal cancer treatment strategy. The antitumor effect of ECI-NPs on common cancer cells has been demonstrated, and further evaluation using resistant cell lines is planned.

**FIGURE 8:**
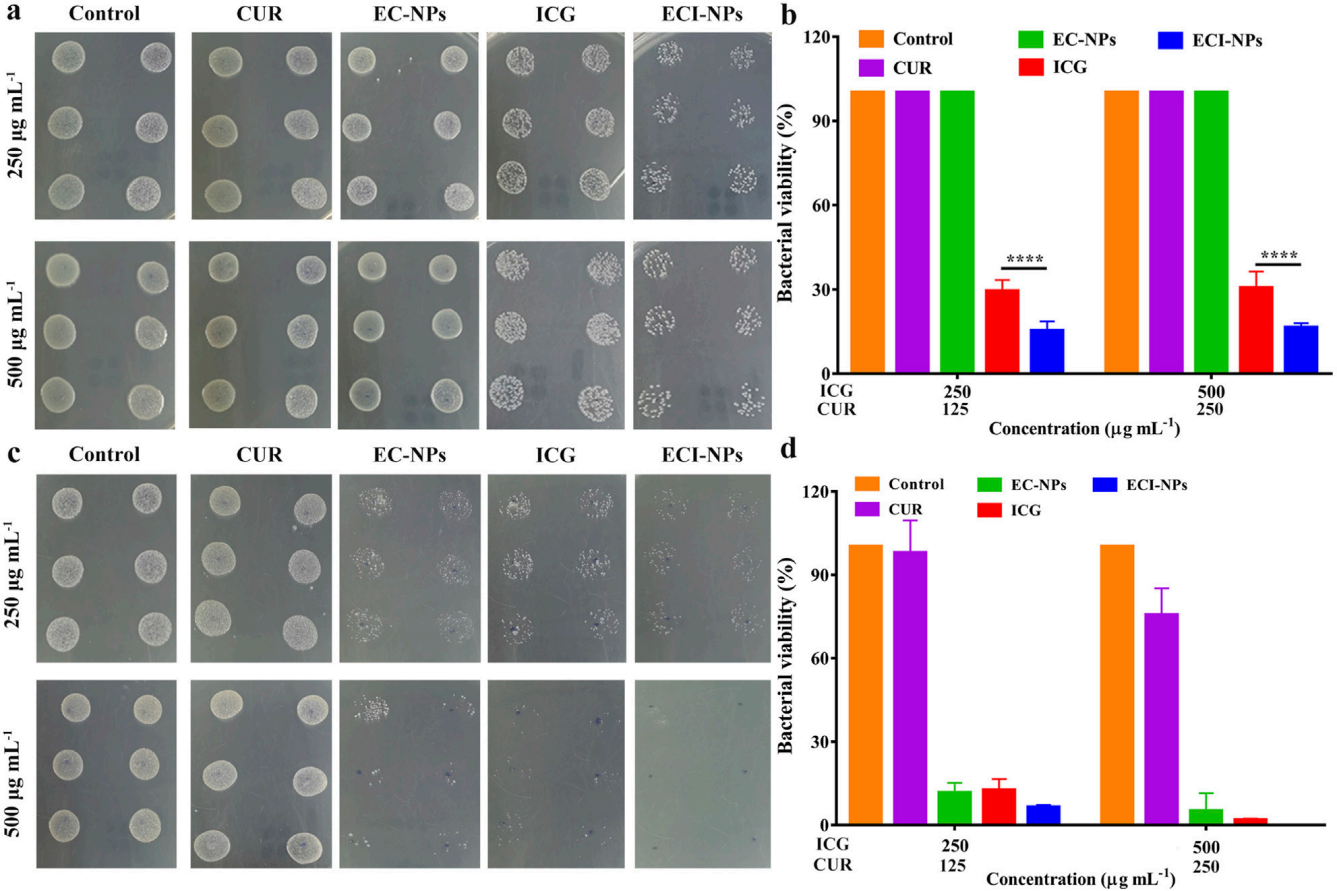
Agar plates digital photographs of **(A)** *E. coli* and **(C)** *S. aureus* after treatment with ECI-NPs. Bacterial viability analysis of **(B)** *E. coli* and **(D)** *S. aureus*, showing the antibacterial activity of ECI-NPs against different pathogens.

To explore the antibacterial activity of ECI-NPs, we selected Gram-negative bacteria (*E. coli*) and Gram-positive bacteria (*S. aureus*) for plate counting assays. Different concentrations of material dispersions were mixed with bacteria and incubated with shaking. After 808 nm laser irradiation, the mixtures were plated on agar plates and incubated for 24 h before counting. As shown in Figures 8A, B, in the *E. coli* antibacterial assay, the colony counts in the CUR and EC-NPs groups were similar to the Control group, indicating that CUR and tea polyphenols alone are insufficient to inhibit *E. coli* proliferation. With increasing concentration, the bacterial viability in the ICG and ECI-NPs groups showed no significant difference. At the same concentration, light exposure resulted in a notable decrease in bacterial viability in both ICG and ECI-NPs groups. The bacterial survival rate in the ICG group was around 30%, significantly lower than in the Control, CUR, and EC-NPs groups, highlighting the superior phototoxic effects in antibacterial treatment. The bacterial survival rate in the ECI-NPs group was approximately half of that in the ICG group, indicating better inhibition of *E. coli* by ECI-NPs.

In the *S. aureus* experiment (Figure 8C), at equivalent concentrations, the colony counts of EC-NPs, ICG, and ECI-NPs were significantly lower than those of the Control and CUR groups, demonstrating the antibacterial activity of these three materials against *S. aureus*. In contrast to the *E. coli* experiment, EC-NPs exhibited notable antibacterial effects. With increasing concentration, the colony counts in the EC-NPs, ICG, and ECI-NPs groups decreased, indicating a concentration-dependent antibacterial activity. Figure 8D shows that CUR at a concentration of 500 μg/mL reduced the viability of *S. aureus*. However, CUR’s antibacterial effect was considerably less than that of EC-NPs. The bacterial viability in the EC-NPs group was 11.44% and 4.91% at 250 μg/mL and 500 μg/mL, respectively, demonstrating significant inhibition of Gram-positive bacteria. After light exposure, the bacterial survival rates in the ICG and ECI-NPs groups at 250 μg/mL concentrations were 12.36% and 6.24%, respectively. The superior antibacterial activity of ECI-NPs is attributed to the chemical activity of CUR and the phototoxicity of ICG. The antibacterial activity of the tea polyphenol nanocarriers warrants further investigation. At a concentration of 500 μg/mL, ECI-NPs completely inhibited bacterial proliferation, with bacterial survival significantly lower than in the ICG group (1.69%). This further underscores the high antibacterial activity of ECI-NPs against *S. aureus*. The antibacterial results indicate that ECI-NPs effectively inhibit the proliferation of both *E. coli* and *S. aureus*, presenting a promising chemotherapeutic-phototherapeutic antibacterial nanomaterial.

## 4 Conclusions and future perspectives

This study successfully developed tea polyphenol-based multifunctional nanocarriers (E-NPs) and optimized their loading of CUR and ICG, demonstrating excellent photodynamic and photothermal properties along with significant anticancer and antibacterial effects. These findings highlight the broad potential of tea polyphenol-based nanomaterials in multi-disease treatment applications. ECI-NPs show promise as novel therapeutic agents in cancer treatment, particularly for targeting refractory or metastatic tumors. Additionally, their application in combating antibiotic-resistant bacterial infections is noteworthy, especially for managing hospital-acquired and chronic infections. The ability of ECI-NPs to provide both anticancer and antibacterial effects suggests their potential for combined therapy in various pathological conditions. Future research should focus on evaluating their performance in clinical trials, including safety, efficacy, and dosage optimization, to facilitate their practical application. These efforts are expected to advance the use of tea polyphenol-based multifunctional nanomaterials in the medical field, improving patient outcomes and quality of life.

## Data Availability

The raw data supporting the conclusions of this article will be made available by the authors, without undue reservation.
